# Rehabilitation in patients with cerebellar ataxias

**DOI:** 10.1590/0004-282X-ANP-2021-0065

**Published:** 2022-02-28

**Authors:** Hsin Fen Chien, Marise Bueno Zonta, Janini Chen, Giovana Diaferia, Celiana Figueiredo Viana, Hélio Afonso Ghizoni Teive, José Luiz Pedroso, Orlando Graziani Povoas Barsottini

**Affiliations:** 1 Universidade de São Paulo, Faculdade de Medicina, Departamento de Ortopedia e Traumatologia, São Paulo SP, Brazil. Universidade de São Paulo Faculdade de Medicina Departamento de Ortopedia e Traumatologia São Paulo SP Brazil; 2 Universidade Federal do Paraná, Hospital de Clínicas, Departamento de Medicina Interna, Serviço de Neurologia, Unidade de Distúrbios do Movimento, Curitiba PR, Brazil. Universidade Federal do Paraná Hospital de Clínicas Departamento de Medicina Interna Curitiba PR Brazil; 3 Universidade Federal de São Paulo, Departamento de Neurologia, Divisão de Neurologia Geral e Unidade de Ataxia, São Paulo SP, Brazil. Universidade Federal de São Paulo Departamento de Neurologia Divisão de Neurologia Geral e Unidade de Ataxia São Paulo SP Brazil

**Keywords:** Cerebellar Ataxia, Rehabilitation, Physical Therapy Specialty, Speech Therapy, Occupational Therapy, Ataxia Cerebelar, Reabilitação, Especialidade de Fisioterapia, Fonoterapia, Terapia Ocupacional

## Abstract

Cerebellar ataxias comprise a heterogeneous group of diseases characterized by motor and non-motor symptoms, which can be acquired, degenerative, or have a genetic cause, such as spinocerebellar ataxias (SCA). Usually, the genetic and neurodegenerative forms of cerebellar ataxias present a progressive and inevitable worsening of the clinical picture so that rehabilitation treatment is fundamental. Rehabilitation treatment includes physical therapy, respiratory therapy, speech, voice and swallowing therapy, occupational therapy, and new technologies, such as the use of exergames. The current treatment of patients with cerebellar ataxias, especially neurodegenerative forms, genetic or not, should include these different forms of rehabilitation, with the main objective of improving the quality of life of patients.

## RESUMO

As ataxias cerebelares compreendem um grupo heterogêneo de doenças caracterizadas por sintomas motores e não motores, que podem ser adquiridas, degenerativas ou ter uma causa genética, como as ataxias espinocerebelares (AEC). Normalmente, as formas genéticas e neurodegenerativas de ataxias cerebelares apresentam uma piora progressiva e inexorável do quadro clínico e o tratamento com reabilitação é fundamental. O tratamento com reabilitação inclui a fisioterapia, fisioterapia respiratória, fonoterapia, terapia ocupacional e novas tecnologias, como o uso de exergames. O tratamento atual de pacientes com ataxias cerebelares, em particular as formas neurodegenerativas, genéticas ou não, deve incluir essas diferentes formas de reabilitação, com o objetivo principal de melhorar a qualidade de vida dos pacientes.

## INTRODUCTION

Ataxia is a neurological disorder characterized by loss of coordination, particularly affecting gait and balance. In general, ataxia may be related to cerebellar disorders (cerebellar ataxia) or peripheral nerve involvement (sensory ataxia). Besides gait disorders, patients with ataxia also commonly present with limb incoordination, oculomotor changes, slurred speech, and dysphagia. Considering the above symptoms, patients with ataxia usually have marked reduction in quality of life that requires rehabilitation programs^1^.

Concerning etiology, ataxia may be acquired or may have a genetic cause. A positive family history guides investigation for genetic forms of ataxia, such as the autosomal dominant spinocerebellar ataxias (SCAs)^1^. Usually, genetic and neurodegenerative forms of ataxia have inevitable progressive worsening. Although several symptomatic treatments have been proposed for patients with progressive ataxias, there is no specific therapy to interrupt disease progression or to recover the cerebellar atrophy^2^.

Some studies suggest that rehabilitation may improve some symptoms, quality of life, and independence in patients with cerebellar ataxia. However, several studies are non-randomized or non-controlled^3^ and new technologies are progressively being described and used, which complicates the analysis of some clinical trials. Thus, it is difficult for neurologists and health care professionals to keep up with advances in rehabilitation.

There are several concerns when a patient with cerebellar ataxia undergoes a rehabilitation program. First, how sustainable is the gain of functional and how does it affect the cerebellar pathways. It is well known that motor learning and adaption may be impaired in patients with cerebellar ataxia and that the cerebellum and cerebellar pathways may be affected by repetitive motor training. And secondly, how effective repetitive and daily activities are in maintaining motor function as the neurodegenerative process progresses ^4^. As far as we are concerned, an intensive rehabilitation program can partially overcome impaired motor function and serve as valuable therapeutic strategy for patients with cerebellar ataxia. 

In this narrative review article, we provide an update on the main rehabilitation interventions for patients with cerebellar ataxia, and the following topics are discussed in details: physical therapy, outcome measures and clinical scales, exergames and new technologies, respiratory rehabilitation, speech, voice and swallowing therapy, and occupational therapy in ataxia.

## METHODS

We searched the following databases for published studies in Portuguese and English over the period 1990-2020: PubMed and Scientific Electronic Library Online (SciELO). The search process included articles that described the intersection between “ataxia” OR “cerebellar ataxia” AND “physical therapy”, “postural balance”, “outcome measure”, “virtual reality”, “respiratory rehabilitation”, “speech therapy”, “dysarthria”, “deglutition and “occupational therapy**”.**

Articles and guidelines describing major points in new technologies, robotic rehabilitation, and exergames were also included. Fifty-eight articles were selected and included in this article.

## PHYSICAL THERAPY

Soon after the diagnosis of ataxia, all individuals should be referred for physical therapy even if they have only mild symptoms. Physiotherapy should explore the patient potential and alleviate their symptoms as much as possible. It should continue throughout all stages of the disease, as rehabilitation can improve the health and well-being in individuals with ataxia^5^.

**Figure 1.**
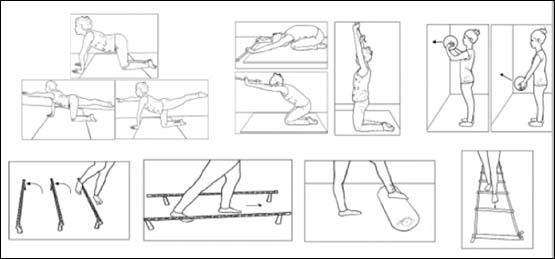
Example of coordination and balance training exercise^59^.

**Figure 2.**
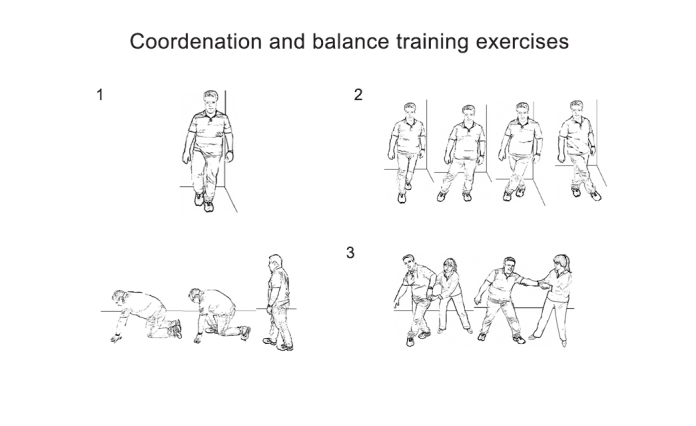
Example of coordination and balance training exercises^60^. 1) Static balance: standing on 1 leg; 2) Dynamic balance: sidesteps; 3) Steps to prevent falling and falling strategies.

Promising results of physiotherapy for patients with ataxia were first reported by Ilg and colleagues^6^. They showed that coordinative training improved motor performance and reduced ataxia symptoms, enabling patients to achieve personally meaningful goals in everyday life. Examples of coordination and balance training exercises are shown in Figures 1 and 2.

(Extracted, with permission, from references 59 and 60).

(Extracted, with permission, from references 59 and 60).

Marquer and colleagues^7^ published the first systematic review on the treatment of postural disorders in cerebellar ataxia in 2014. The authors selected nineteen articles, of which three were randomized controlled trials (RTC), covering various etiologies of cerebellar ataxia. They concluded that there was moderate level of evidence that rehabilitation was efficient in improving postural capacities of patients with cerebellar ataxia, particularly in those with degenerative ataxia or multiple sclerosis. The evidence was clear in the case of intensive rehabilitation programs but still weak for techniques such as virtual reality, biofeedback, and treadmill exercises with body weight or torso support. Recovery of motor function was the subject of a review study by Synofzik and Ilg^8^ published in the same year. Encouraging results were reported and demonstrated that high-intensity motor coordination training offered a significant benefit in patients with degenerative ataxia, with gains in stability and motor coordination.

Milne and colleagues^9^ published in 2017 a systematic review on rehabilitation interventions specifically for individuals with genetic degenerative ataxia. They also investigated long-term outcomes from rehabilitation and optimal duration and intensity of rehabilitation. Seventeen studies met their eligibility criteria, five were RTC, but the majority of the studies were classified as level III or IV. The total sample in their studies was 148 patients with autosomal dominant ataxia and 85 with autosomal recessive ataxia. Rehabilitation interventions included coordination and balance training, multifaceted inpatient rehabilitation, a cycling regime, balance exercises with technology-assisted biofeedback, respiratory muscle training, and treadmill training. The authors concluded that there was consistent evidence that rehabilitation improves function, mobility, ataxia, and balance. Less than half of the studies included in their review assessed long-term outcomes and their follow-up time frame varied considerably. 

The following year, Zesiewic and colleagues^10^ reviewed the literature on treatment of cerebellar motor dysfunction, covering pharmacologic, surgical, or other interventional therapies. They cited the Miyai and colleagues (2012)^4^ Class I study showing that rehabilitation improves ataxia and function, with acceptable safety and tolerability. They also included a Class II study^11^ reporting that transcranial stimulation possibly improves cerebellar motor signs. The authors reasoned that rigorous studies with ataxia patients are challenging since most of the published articles were Class IV rehabilitation studies. They also stated that other therapies that are outside the scope of their review could have clinical value even without having the evidence of a clinical trial. 

In 2019, Hartley and colleagues^12^ published a systematic review in which they evaluated the range, scope, and methodological quality of studies investigating the effectiveness of exercise and physical therapy interventions for children with ataxia. From a total of 1988 studies, twenty were included in the review. The studies reported promising results but most of them had low methodological quality, used small sample sizes, and were heterogeneous in terms of interventions, participants and outcomes. Therefore, the authors could not give firm conclusions about the effectiveness of exercise and physical therapy for children with ataxia. 

The evidence suggests that rehabilitation improves function, mobility, ataxia, and balance in adults and provides promising results for children. Still, we need adequately powered clinical trials to confirm the beneficial effects of specific interventions, define their optimal frequency and intensity, and determine the time of follow up. Clinical trials should include a more homogeneous sample of patients for different diseases and patients in prodromal or different disease stages. 

Knowing that coordination and balance exercises are beneficial for ataxic patients, we face the challenge of: 1. finding ways to adapt the routine of patients and keep them engaged in a continuous, varied, and effective training; 2. adapting training to the specific needs of each patient according to disease stage, age, and interests; 3. keeping patients in intensive exercise practice; 4. designing studies with long-term follow-up; and 5. integrating the rehabilitation program into clinical practice^13^.

Coordination and balance exercises or other exercise modalities, as well as new interventions (such as transcranial magnetic stimulation) need to be studied with higher methodological quality to confirm or refute their indication and to determine the best practice for the treatment of individuals with ataxia.

## OUTCOME MEASURES AND CLINICAL SCALES

The rehabilitation of patients with ataxia involves a thorough assessment to establish the patient’s current level of functioning and to set up treatment goals and strategies. The quantification of ataxia severity is very important for both clinical practice and research as it allows a better assessment of the effect of rehabilitation. Ataxia can be assessed using different validated instruments such as those cited below. 

 The Scale for the Assessment and Rating of Ataxia (SARA)^14^^,^^15^ is the most frequently used scale to monitor progress of cerebellar ataxia, especially during the rehabilitation process. It is a simple, quick, and easy to use tool consisting of eight items, with accumulative score ranging from 0 (no ataxia) to 40 (most severe ataxia), addressing gait, posture, balance, speech disturbance, coordination and diadochokinesia. 

 In the study by Bourcier et al. (2020)^16^, SARA demonstrated adequate content validity with possible influence of pyramidal and/or neuropathic involvement. It also demonstrated excellent construct validity (*r*_*s*_ = 0.77-0.95) and internal consistency (Cronbach's α = 0.89). 

The International Cooperative Ataxia Rating Scale (ICARS)^17^^,^^18^ comprises 19 items divided in four subscales: posture and gait disturbances, kinetic functions, speech disorders and oculomotor disorders, along with a functional test (Archimedes spiral). The maximum cumulative score is 100 points. ICARS presents high inter-rater reliability (ICC: 0.95), high test-re-test reliability (ICC: 0.97), adequate internal consistency (Cronbach’s α: 0.94) and good internal structural validity^19^. 

The Neurological Examination Score for Spinocerebellar Ataxias (NESSCA) is another reliable measure based on a standardized neurological examination, composed of 18 items that yield a total score ranging from 0 to 40. It was initially developed to be used for Machado Joseph disease^20^. Its interrater reliability ranges from 0.8 to 1 across individual items (P < 0.001); internal consistency, indicated by Cronbach’s α, is 0.77. NESSCA scores significantly correlate with measures of disease severity: disease stage (rho = 0.76, P < 0.001), duration (rho = 0.56, P < 0.001), and length of CAG repeat (rho = 0.30, P < 0.05). This scale comprises a wide range of neurological manifestations and can be also useful for other SCAs, provided that validation standards are demonstrated, like it was for SCA2^21^. 

Two quantitative performance measures or timed tests, described below, are used to supplement the results of clinical rating scales.

The Spinocerebellar Ataxia Functional Index (SCAFI)^22^ is composed of a timed 8-meter walk at maximum speed, the 9-hole peg test (9HPT), and a speech performance assessment called PATA rate (it refers to how often the subject can repeat the syllables “PATA” within ten seconds). SCAFI was validated in a large multi-center cohort of SCA patients^23^.

The Composite Cerebellar Function Severity Index (CCFS)^24^ score was validated in adults and children with ataxia. The assessment is based on the combined time to perform two tasks: 9HPT and a click test. Both tasks include a series of alternative movements: placing pegs and finger-pointing cycles, adjusted for age. The test is easy to perform and correlates with severity of cerebellar impairment. 

There are some clinical balance assessment tools that can be used to quantify the severity of postural disorders in cerebellar ataxia. The Berg Balance Scale (BBS) and the Timed Up and Go test (TUG) were identified as the best outcome measure and they have at least 75% inter rater reliability among the experts^25^. The TUG is also considered useful as a generic gait assessment, as well as the 6-minute Walk Test and the gait speed over 10 m, which provide quantitative estimates of walking ability^7^. 

In addition to the clinical scales, functional scales are extremely important in the assessment of patients with ataxia, considering that the expected improvement should impact the patient's daily life. Although the concept of the International Classification of Functioning, Disability and Health (ICF)^26^ could probably improve the prioritization of problems, it is still underused in patients with SCA. The application of ICF in daily practice requires the use of several basic sets that are specified for the disease, but none is available for SCAs. The scales mentioned above focus mainly on the functional aspects of each individual, addressed in ICF as “Body structure and Body function”. Analysis with the ICF may help health professionals to identify the “Activity and Participation” aspects of SCA, especially on mobility function^27^. Future questionnaires should be developed using ICF standards for better assessment of ataxic patients.

## EXERGAMES AND NEW TECHNOLOGIES

Technologies are a complementary resource to expand ataxia rehabilitation. Studies have demonstrated that new intervention methods can improve the patient's quality of life, and adding different technology devices in patient training may improve range of motion, promote motor challenges, improve balance, and recover motor function in patients with ataxias^8^^,^^28^^,^^29^.

An emerging field of literature and research is the use of virtual reality (VR) and augmented reality as therapeutic and rehabilitation modalities^30^. These technologies are accessible, ludic, and motivational, and use body motion sensors on platforms and wearable sensors to capture and analyze patients’ movements. It can be easily incorporated into physical therapy programs but also be implemented in the routine activity of the patient. 

 Exercises through video games are called exergames. This training modality usually involves multi coordination, weight transfer, and balance exercises that might promote motor function in ataxias. There are a few clinical studies reporting the efficacy of exergames in cerebellar ataxia, but this VR training has shown functional capacity advantages in neurorehabilitation programs for post-stroke upper limb recovery, as well as for degenerative and vascular-related cognitive deficits. Videogames also enhance oculo-motor coordination, anticipatory capacity, and rapid movements executed in a virtual environment^28^.

The review by Synofzik and colleagues^8^ (2014) indicates that exergame-based balance and coordination training might benefit patients with ataxia. It is easy to practice at home and it increases long-term adherence. A home-based study demonstrated that young patients with severe degenerative ataxia in advanced stage of their disease could benefit from individualized videogame training. After 12 weeks of coordinated trunk and postural control when playing exergames, the functional capacity and activities of daily living improved.^31^ Nintendo^®^ Wii exergames may also be a potential tool for improving the functional capacity of degenerative ataxia patients. Patients training with four games (Soccer Heading, Tightrope Walk, Table Tilt, and Ski Slalom) for one 50-min session for 20 days, using a hand-held remote and a Wii balance board system, resulted in postural sway improvement. Improvements in balance and gait, fall frequency, and self-confidence were also observed^32^.

Exergames may also be associated with audio-biofeedback integrating additional sensory modalities to compensate for deficient postural control. A sensor captures trunk acceleration and sends this information to a smartphone that plays an acoustic feedback during exergame training period. Although the effects of long-term training outcomes are not yet known, the preliminary study shows that acoustic sensory information could compensate for the impairment in proprioceptive and vestibular signals to reduce postural sway^33^.

Patients with mild symptoms may benefit from exergames according to a recent review on different training programs for SCA rehabilitation. For more severe cases, the game modality should target prevention of falls, improvement of mobility, resistance, posture, balance, and muscular strength. In later stages, patients should play the game with postural adjustments, and a treadmill may be combined into the videogame training to improve balance and gait^28^.

A VR treadmill with a motion capturing system allows analyses of spatial, temporal, kinematic, and kinetic parameters of consecutive steps in real time. Furthermore, VR-enhanced gait training is an effective method to improve spatiotemporal and functional parameters in persons with movement disorders of the central nervous system ^34^. One study using VR and dual-belt treadmill integrated with a two-force platforms synchronized with a projected environment, demonstrated that training with immersive VR is a promising approach for ataxic gait rehabilitation, even in chronic conditions^35^. An alternative way to assess gait is to incorporate a portable sensor into the patient’s daily life routine. Despite the high variability of gait analysis of individuals with ataxia, one study demonstrated that real-life gait assessment correlated with the clinical severity of ataxia in patients with degenerative cerebellar disease^36^. 

The Kinect^®^ sensor, a low cost camera system that captures and analyzes movements in three dimensions, can accurately measure timing and gross spatial characteristics of clinically relevant movement disorders^37^. Based on these considerations, Wang and colleagues^29^ developed a coordinative training and balance-based exergame program using the Kinect^®^ sensor. Patients with SCA3 participated in a 4-week, 40-minute and thrice-weekly randomized trial addressing reaching task. After training, participants in the exergaming group had a significant decrease in the total SARA score and in the gait-posture SARA subscore. On the other hand, Summa and colleagues^38^ developed a new assessment tool called SARA Home. It was based on the SARA’s scale structure adapted into a VR interface and integrated Kinect cameras, leap motion controllers, and Kinect microphones to track and quantify gait, hand movements, and speech. Their study highlighted the feasibility and acceptability of the system, suggesting a potential use in clinical practice.

Robotic rehabilitation systems have the potential to measure abilities and serve as therapeutic tools. They can assess and measure motor abilities, posture and limb position, strength, gait, and balance. Using a computerized visual robotic arm system, one study demonstrated that such device could quantify changes in arm trajectories and differentiate healthy controls from Friedrich ataxia patients when they performed a point-to point upper limb movement task. The subjects with ataxia exhibited slower movements, lower accuracy, and less smoothness. Moreover, the robotic indices were directly correlated with SARA^39^. Although promising results were described in neurological rehabilitation with robotics, few are related to ataxia. A case report of a young patient with Friedrich ataxia who underwent 24 intensive sessions with the Lokomat^®^ robotic device combined with cerebellar transcranial direct current stimulation, showed a significant improvement of functional capacity after the training program^40^. Future clinical studies with larger sample sizes are required to evaluate the efficacy and effectiveness of robotics rehabilitation of ataxic patients.

## RESPIRATORY REHABILITATION

Few studies report the effects of physical therapy on respiratory complications of ataxic patients. Restrictive lung disease is common in Ataxia Telangectasia (AT) and is characterized by lower forced vital capacity. The bulbar degeneration and congenital immunodeficiencies may contribute to the susceptibility to chronic respiratory and pulmonary infections. Other factors also influence the development of pulmonary disease in AT including premature aging, inflammation, oxidative stress, and an inability to properly repair damage that occurs in the lungs over time^41^. Children and adults with increased bronchial secretions may benefit from routine chest therapy using the manual method and a cappella device or a chest physiotherapy vest. Chest physiotherapy can help eliminate mucus from the lower bronchial tree. However, an adequate cough is needed to remove the secretions. In people who have decreased lung reserve and a weak cough, the use of an insufflator-exsufflation device may be useful as a maintenance therapy or during acute respiratory illnesses to remove bronchial secretions from the upper airways.

Felix and colleagues^42^ conducted a 24-week inspiratory muscle training program with 11 patients with AT and nine healthy volunteers. The study included the following assessments: ventilometry, subjective sensation of dyspnea, maximal inspiratory pressure (MIP), maximal expiratory pressure (MEP), and the SF-36 quality of life questionnaire. The load used was set at 60% of the MIP, and the training was performed for 20 min daily. Patients with AT showed a significant improvement on sensation of dyspnea, ventilatory pattern, lung volume, respiratory muscle strength, and on the health and vitality domains of SF-36 questionnaire after the end of the intervention.

Patients with SCA1, SCA2, and SCA3 may present restrictive pulmonary dysfunction and upper airway obstruction. The pulmonary dysfunction in SCA is due to a lack of muscle coordination and inability to sustain respiratory effort ^43^. A recent study with SCA2 patients suggested that ventilatory dysfunction, even asymptomatic, is related to increased imbalance, functional dependence, and worse SARA score^44^. Ventilatory support, assisted cough, and air-stacking technique should be prescribed to these patients as needed to relieve their symptoms.

Inspiratory muscle weakness, especially in the diaphragm, causes ventilatory disorders during sleep, including hypoventilation/hypercapnia leading to fatigue, daytime sleepiness, and worse sleep quality in ataxia^45^. There are no studies reporting respiratory muscle training (RTM) in ataxic patients, but such training is applied to amyotrophic lateral sclerosis (ALS) and Duchenne muscular dystrophy (DMD) patients with some improvement in pulmonary function. Although the results in the few studies published to date on ALS and DMD^46^, it would still be worthwhile to investigate the effect of RTM in ataxia. 

Because there are few studies on ventilatory and respiratory rehabilitation in ataxia, we lack rational guidelines for the treatment of pulmonary complications in such population. More studies are needed to address this important issue in the near future.

## SPEECH, VOICE AND SWALLOWING THERAPY

Ataxia affects speech and swallowing, therefore speech-language interventions are essential because dysarthria affects normal socialization. Voice and verbal communication affects education, work, social functioning and expression, and patients with dysphagia are at risk of repeated aspiration and subsequent pneumonia^13^. Referral for dysphagia and dysarthria rehabilitation as early as possible is also very important as both symptoms and quality of life (QoL) perceptions can be improved by speech-language therapy^47^.

Interventions targeting mechanical and functional components of swallowing and speech articulation are most effective when these impairments are detected and understood. Studies on changes in speech/voice and swallowing in degenerative ataxias are scarce, but identifying peculiar markers of SCA presentations may help therapists to intervene early on the patient’s specific needs. Some studies on degenerative ataxias show different symptoms according to the mutation carried by the patient. 

Jardim and colleagues^48^ evaluated 62 patients with SCA3 presenting dysarthria, muscle fasciculation, pyramidal syndrome, and ophthalmoplegia. They found that the earlier the age of onset, the more severe the progression of SCA3, and the later the age of onset, the less severe the progression of disease. Same findings were also noted by other authors^49^^,^^50^. Vogel and colleagues^51^ observed that dysarthria and swallowing deficit could be correlated to disease severity and progression in SCA. In a series of SCA2 patients who underwent a comprehensive assessment battery, slower speech rate was already observed in early stage ataxic patient, whereas dysphagia was found in both pre-ataxic and ataxic SCA2 patients.

The best intervention also requires proper assessment of changes in speech and voice characteristics. Wolf and colleagues^52^ studied a group of SCA3 patients and observed that they had imprecise articulation with slow rate of speech, hoarse-breathy voice quality, and decreased loudness. Moreover some patients had also pyramidal or extrapyramidal involvements determining variations in voice qualities. When extrapyramidal symptoms are predominant, voice characteristics may be similar to that of Parkinson's disease and include reduced loudness, monopitch, hoarseness, and a breathy voice quality. According to the author, the dysarthria in patients with SCA3 is more similar to mixed than to ataxic dysarthria. They also emphasized that complaints involving communication may not be consistent with objective findings in the clinical evaluation, and thus speech therapists must pay special attention to patients’ expectations and communication skills.

Dysphagia is common in individuals with ataxia. An epidemiological, clinical, and pathological study on SCA3 reported that dysphagia usually occurs after 8 years of disease onset in 70% of patients, and after 15 years it becomes moderate or severe and may cause death because of tracheobronchial aspiration, bronchopneumonia, or malnutrition^53^. Patients with degenerative ataxias have greater difficulty swallowing liquids than solid foods, and penetration is significantly higher for liquids than solid foods^54^. Therefore, early intervention to improve oral motor control and adapt food viscosity and bolus volume^49^^,^^52^^,^^55^ may increase patients QoL and survival. 

Further studies on speech articulation and swallowing may improve our knowledge on degenerative or other causes of ataxia. Early rehabilitation may also maintain the best possible communication at each stage of the disease, preserve social interaction, and increase swallowing safety to prevent complications related to aspiration and malnutrition.

## OCCUPATIONAL THERAPY IN ATAXIA

Few studies have explored the effect of occupational therapy (OT) in ataxic patients and most have low to moderate level of quality. Despite such limitations, a recent systematic review has shown that conventional OT can improve balance and coordination of patients with degenerative ataxia^56^. One study showed that patients with SCA who underwent six months of OT improved their Hamilton depression score indicating that even if no effect is observed in short-term therapy, patients’ mood may benefit from this intervention^57^.

Combined intensive occupational and physical therapy seems to be more effective then OT intervention alone. Miyai et al.^4^ reported that after a 4-week intensive rehabilitation program in inpatients with diverse cerebellar ataxia pathology not only improved their SARA, gait speed, and activity of daily living (ADL) scores, but these gains were maintained after 12 weeks of the training completion. Another trial assessed the efficacy of a 24-week combined intensive rehabilitation program in SCA2 patients. After the completion of the treatment, patients had a significant improvement in motor cerebellar symptoms, balance, and limb coordination. The authors suggest that the improvement observed in this degenerative condition is likely because of partial preservation of motor learning and motor plasticity mechanisms, which emphasize that rehabilitation could control the progression of the disease^58^.

OT should be prescribed and adapted to the specific needs of the patient with ataxia. Although the benefits of this intervention have been described, many issues remain unanswered such as: how long, how frequent, how intense, and what modalities should be prescribed. Besides, retention benefits may be influenced by the continuity of home exercise or adherence to therapies in outpatient facilities. Adding new therapies or other electronic devices such as exergames or Kinect may contribute to increase the adherence and interest of younger patients. Lastly, a better understanding of symptoms and prodromal signs of each degenerative ataxia may help identify the patient’s specific needs and design a better OT rehabilitation program (Table 1).

**Table 1.** Summary of studies on rehabilitation of ataxia patients.

	Study population/ Sample size	Type of study	Intervention/ Tecnhique	Authors
Physiotherapy	Progressive ataxia (N=16)	Experimental study	Intensive coordinative training improves motor performance	Ilg et al., 2009^6^
	Degenerative ataxia or multiple sclerosis	Systematic review	Intensive rehabilitation improves postural capacity and motor coordination	Marquer et al., 2014^7^
	Degenerative ataxia	Review article	Intensive rehabilitation improves stability and motor coordination	Synofzik and Ilg, 2014^8^
	Genetic degenerative ataxia	Systematic review	Coordination and balance training, multifaceted inpatient rehabilitation, cycling, balance exercises with technology assisted biofeedback, respiratory muscle training, and treadmill	Milne et al., 2017^9^
	Children with ataxia	Systematic review	Insufficient data to support or refute effectiveness of exercise and physical therapy for children with ataxia	Hartley et al., 2019^12^
Outcome measures and clinical scales	Cerebellar ataxia	Validation	Scale for the Assessment and Rating of Ataxia (SARA)	Trouillas et al.,1997^17^
	Spinocerebellar degeneration (N=27), Spinocerebellar subtypes (N=91)	Validation	International Cooperative Ataxia Rating Scale (ICARS)	Yabe et al., 2008^14^
	SCA3	Validation	The Neurological Examination Score for Spinocerebellar Ataxias (NESSCA)	Kieling et al.,2008^20^
	SCA	Validation	SCA Functional Index (SCAFI)	Schmitz-Hübsch et al., 2008^22^
	Autosomal dominant cerebellar ataxia Autosomal dominant spastic paraplegia	Validation	Composite cerebellar functional severity score (CCFS)	du Montcel et al., 2008^24^
	Cerebellar ataxia	Observational study	Recommended outcome measures for postural disorders severity screening (BBS and TUG)	Winser et al., 2015^25^
Exergame and New Technologies	Cerebellar ataxia	Review	Exergame for SCA rehabilitation	Lanza et al., 2019^28^
	Friedreich’s Ataxia (N=14)	Observational study	Robotic devices for clinical measures and ataxia rehabilitation	Germanotta et al., 2017^39^
Respiratory Rehabilitation	Ataxia Telangectasia (N=11)	Experimental study	Inspiratory muscle training	Felix et al., 2014^42^
	Spinocerebellar ataxias (N=30)	Experimental study	SCA may present restrictive pulmonary dysfunction and upper airway obstruction.	Sriranjini et al., 2010^43^
Speech language therapy	SCA 3 (N= 33)	Observational study	Early intervention for oral motor control, changing food viscosity and bolus volume	Wolf 2008^52^
Occupational therapy	Degenerative ataxia	Systematic review	For balance and coordination	He et al., 2020^56^
	Spinocerebellar ataxia type 3 (N=26)	Experimental study	Mood improvement	Silva et al., 2010^57^

SARA: Scale for the Assessment and Rating of Ataxia; ICARS: International Cooperative Ataxia Rating Scale; BBS: Berg balance score; TUG: Timed up and Go test; SCA: Spinocerebellar ataxia.

In conclusion, cerebellar ataxias are a heterogeneous and complex group of disorders characterized by motor and non-motor symptoms that poses major challenges to neurologists and other health providers involved in patient care and rehabilitation. While there is no curative treatment for the vast majority of both genetic and acquired cases of ataxia, rehabilitation including motor and respiratory physical therapy, speech therapy, and occupational therapy is an essential component of patient care.

We should always keep in mind that many of the problems that cause patient discomfort and affect their quality of life include not only cerebellar symptoms, but also other complaints that have not been extensively studied in patients with ataxia, such as dysphagia, breathing difficulty, pain, spasticity, and cramps.

Although the level of evidence of articles on rehabilitation of patients with ataxia are not usually high, there is almost a consensus among specialists that early rehabilitation is beneficial for these patients in long-term. Further investigations are definitely needed to better determine the most effective rehabilitation approaches and which patients are likely to benefit from earlier and more intensive rehabilitation.^59^^,^^60^

This review highlights the importance of multidisciplinary care for patients with ataxia, and health services should ensure appropriate and humanized care for these patients.
